# Optimisation of methods for bacterial skin microbiome investigation: primer selection and comparison of the 454 versus MiSeq platform

**DOI:** 10.1186/s12866-017-0927-4

**Published:** 2017-01-21

**Authors:** Madhura Castelino, Stephen Eyre, John Moat, Graeme Fox, Paul Martin, Pauline Ho, Mathew Upton, Anne Barton

**Affiliations:** 1 NIHR Manchester Musculoskeletal Biomedical research Unit, Central Manchester Foundation Trust, Manchester Academic Health Sciences Centre, Manchester, England; 2 Arthritis Research UK Centre for Genetics and Genomics, Centre for Musculoskeletal Research, Institute of Inflammation and Repair, University of Manchester, Stopford Building, Oxford Road, Manchester, M13 9PT UK; 3 Microbiology and Virology Unit, Institute of Inflammation and Repair, The University of Manchester, Stopford Building, Oxford Road, Manchester, UK; 4 DNA Sequencing Facility, Faculty of Medical & Human Sciences, The University of Manchester, Stopford Building, Oxford Road, Manchester, UK; 5 School of Biomedical and Healthcare Sciences, Plymouth University Peninsular Schools of Medicine and Dentistry, Plymouth, UK

**Keywords:** Skin microbiome, Bacterial microbiome, Low biomass, 16s rRNA gene, Miseq platform, Next generation sequencing, Methods, Optimisation, Primer selection

## Abstract

**Background:**

The composition of the skin microbiome is predicted to play a role in the development of conditions such as atopic eczema and psoriasis. 16S rRNA gene sequencing allows the investigation of bacterial microbiota. A significant challenge in this field is development of cost effective high throughput methodologies for the robust interrogation of the skin microbiota, where biomass is low. Here we describe validation of methodologies for 16S rRNA (ribosomal ribonucleic acid) gene sequencing from the skin microbiome, using the Illumina MiSeq platform, the selection of primer to amplify regions for sequencing and we compare results with the current standard protocols..

**Methods:**

DNA was obtained from two low density mock communities of 11 diverse bacterial strains (with and without human DNA supplementation) and from swabs taken from the skin of healthy volunteers. This was amplified using primer pairs covering hypervariable regions of the 16S rRNA gene: primers 63F and 519R (V1-V3); and 347F and 803R (V3-V4). The resultant libraries were indexed for the MiSeq and Roche454 and sequenced. Both data sets were denoised, cleaned of chimeras and analysed using QIIME.

**Results:**

There was no significant difference in the diversity indices at the phylum and the genus level observed between the platforms. The capture of diversity using the low density mock community samples demonstrated that the primer pair spanning the V3-V4 hypervariable region had better capture when compared to the primer pair for the V1-V3 region and was robust to spiking with human DNA. The pilot data generated using the V3-V4 region from the skin of healthy volunteers was consistent with these results, even at the genus level (Staphylococcus, Propionibacterium, Corynebacterium, Paracoccus, Micrococcus, Enhydrobacter and Deinococcus identified at similar abundances on both platforms).

**Conclusions:**

The results suggest that the bacterial community diversity captured using the V3-V4 16S rRNA hypervariable region from sequencing using the MiSeq platform is comparable to the Roche454 GS Junior platform. These findings provide evidence that the optimised method can be used in human clinical samples of low bacterial biomass such as the investigation of the skin microbiota.

**Electronic supplementary material:**

The online version of this article (doi:10.1186/s12866-017-0927-4) contains supplementary material, which is available to authorized users.

## Background

The existence of commensals is well established, with the term ‘human microbiome’ being used to describe the sum of all the micro-organisms including bacteria, fungi, viruses, archae and eukaryotes that live in or on a human host at a given time [1]. In recent years, as a result of initiatives such as the NIH Human microbiome project, the scientific community has made great progress in cataloguing these micro-organisms [2–5]. One of the most common sites for investigation is skin, due to the ease of sampling and the potential role of the skin microbiome in the aetiology of skin diseases, such as atopic eczema and psoriasis [6–9]. A challenge in studying the skin microbiome is that healthy skin generally harbours a low microbial biomass; several amplification steps are, therefore, required before sufficient starting material is available for the commonly used DNA sequencing approaches and this increases the risk of introducing sequence artifacts (e.g. chimeric DNA fragments) or detecting contamination from the environment or reagents used [10].

Early studies used amplification, sub-cloning and Sanger sequencing of the highly conserved 16S rRNA gene, which can provide sequence information over the entire length of the 16S rRNA gene in a single reaction. Although this is still the most comprehensive method of bacterial identification, it is expensive and time consuming. Next generation sequencing (NGS) technologies make it possible to carry out identification of members of the microbial community at a much lower cost and with a higher with a higher throughput. Although some regions in the 16S rRNA gene sequence are highly conserved within bacterial species, nine hypervariable regions can be used to determine the identity of different species or genera of bacteria; amplification of short read lengths across these hypervariable regions are sufficient for taxonomic assignment of bacteria [11] to the genus level. Hence, NGS platforms provide an ideal technology for studies of the microbiome despite the fact that the current NGS platforms are only able to generate relatively short read lengths (modal read lengths of 150 to 518 base pairs (bp)) [12, 13]. However, it is important to select the most appropriate hypervariable region for each sample type being analysed, as ‘primer bias’ [14] (selective loss of bacterial groups due to the inability to accurately sequence/identify certain bacteria), may limit the diversity of the bacterial species that can be identified in a sample. As bacterial diversity is variable across different niches, careful selection of the primers for 16S amplicon generation is essential so that the particular hypervariable region sequenced results in the most comprehensive capture of the microbial diversity for the niche under investigation [15].

Until recently, much of the NGS work on the human microbiome was performed using the Roche 454 NGS platform, which offers up to 1000 bp reads. However, increasingly, microbiome studies are utilising newer NGS platforms, such as the Illumina MiSeq, which have been reported to be more cost effective and accurate, but have shorter read lengths [13, 16]. The V4 hypervariable region is traditionally selected for work on the MiSeq as it provides adequate information for taxonomic classification of microbial communities and has demonstrated a lower error rate on the Illumina platform [17]. However, to accommodate the current MiSeq protocol (250 bp paired end sequencing), a longer region (~500 bp) of the 16S rRNA gene was considered for the current work. Our aims were, first, to determine the hypervariable region which will provide the most comprehensive capture of the microbial diversity in human skin communities; second, to confirm that the primer set for amplification of that hypervariable region is specific and will not amplify human DNA contaminants and finally, to compare the performance of the MiSeq with the Roche 454 platform, which has been the established technology. A low biomass mock bacterial community was used to test, optimise and develop the protocol. Skin swabs from healthy volunteers were also tested to confirm the method is applicable to the study of the human skin microbiome.

## Results

### Bacterial DNA extraction using the MoBio PowerSoil DNA isolation kit

We modified the MoBio PowerSoil® DNA Isolation kit manufacturer’s protocol to include and extend the bead beating and heat incubation steps (see Methods) to increase the efficacy of lysis of Gram positive bacterial cell walls. A mock bacterial community (Table 1) was prepared consisting predominantly of Gram positive bacteria, including strains representative of known bacterial skin microbiota. Bacterial DNA was extracted using the modified DNA extraction protocol. Adequate quantities of DNA were obtained (>5 ng/μl), even for traditionally difficult species (e.g. *Staphylococcus* species including *Staphylococcus epidermidis* RP62A), thus validating use of the extraction method.

**Table 1:** DNA yield when using the modified DNA extraction protocol

No	Bacterial Strain	Gram stain	Yield ng/μl
1	*Pseudomonas aeruginosa*–PA01	Negative	17
2	*Bacillus subtilus*–NCTC 831	Positive	34.1
3	*Escherichia coli*–NCTC 8809	Negative	>60
4	*Listeria monocytogenes*–NCTC 2166	Positive	28.7
5	*Mycobacterium phlei*–NCTC 8151		>60
6	*Staphylococcus aureus*–NCTC 7447	Positive	36.2
7	*Enterococcus faecalis* NCTC 370	Positive	40.4
8	*Staphylococcus epidermidis* Rp62a	Positive	8.68
9	*Bordetella bronchiseptica*–253	Positive	>60
10	*Peptoclostridium difficile–*630	Positive	8
11	*Staphylococcus saprophyticus*–NCTC 7687	Positive	23.1

### Selection and optimisation of the primer pairs

Initially, three primer pairs (V1-V3: 63 F/519R [18]; V3-V4: 347 F/803R [19] and V4-V5: 517 F/926R [19]) were selected, all with comparable amplicon length (400 bp-500 bp).

**Fig. 1:**
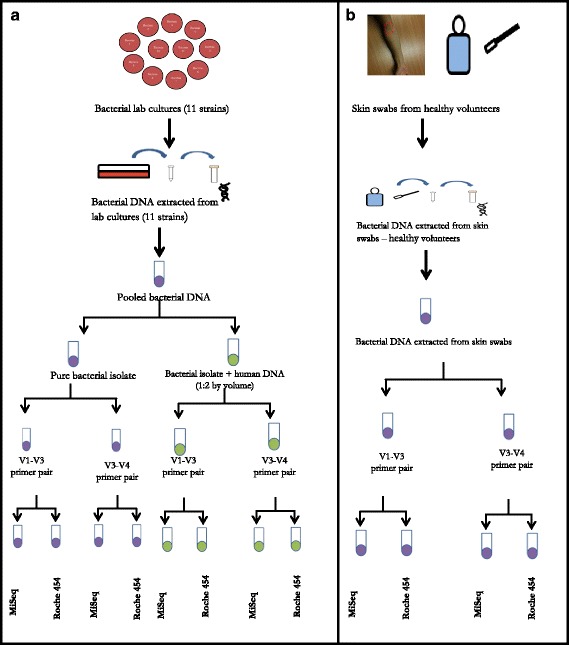
**a** Workflow for the validation work on the MiSeq and the Roche 454 NGS platform using positive control: mock bacterial community. **b** Workflow for the validation work on the MiSeq and the Roche 454 NGS platform using skin swabs: healthy volunteer samples

Two aliquots (100 μl) of low concentration (1.42 ng/μl) mock bacterial DNA community were prepared. One of the aliquots was spiked with human DNA (2 ng/μl concentration) (2:1 bacteria: human DNA by volume) to determine the effect of simulated human DNA contamination on sequencing reads. During PCR optimisation and validation the V4-V5 primer pair amplified human DNA and the primers were, therefore, eliminated from the subsequent workflow. For the remaining primer pairs [V1-V3 (63 F and 519R) and V3-V4 (347 F and 803R)], a single band was visualised after PCR amplification of the human DNA spiked and pure bacterial mock DNA communities. These were then taken forward for re-sequencing, along with human volunteer (HV) skin swab samples, as shown in the workflow (Fig. 1).

### Comparison of the data obtained from Roche 454 and MiSeq platforms

#### Samples and quality control

For the cross-validation of the platforms, skin swabs from two healthy volunteers were used. Two skin swabs (one from the right and another from the left cubital fossa) were obtained from each healthy volunteer. Bacterial DNA extracted from each skin swab was amplified for the V1-V3 and the V3-V4 regions resulting in the generation of 4 amplicon libraries from each healthy volunteer. In addition, the mock bacterial community was divided into two aliquots of which one had simulated human DNA contamination and the other consisted of only the bacterial DNA. These were also amplified for the V1-V3 and the V3-V4 regions of the 16S rRNA gene to generate four amplicon libraries. Twelve samples (4 mock communities and 8 amplicon libraries generated from the four healthy volunteer skin swabs from two individuals) were sequenced by both the Roche 454 Junior and MiSeq platforms.

A total of 5.6 Gb data was generated during the MiSeq run with 85% of the data passing Q30. From the Roche 454 Junior run >650 Mb data was generated with 53% of the data passing Q25. After quality control, denoising, merging of paired end reads for the MiSeq and chimera removal, the MiSeq yielded 5,755,162 reads, at an average length of 264.5 bp (paired reads), whilst the Roche 454 Junior produced 13,305 reads, at an average length of 374.2 bp. The phylogenetic analysis of the data was performed using QIIME [20] software and operational taxonomic units (OTU) were assigned at 97% identity at genus level using the Greengenes database [21]. Three of the eight HV skin swab samples passed stringent QC thresholds on both the MiSeq and Roche 454 Junior, and were available for subsequent NGS platform comparison.

#### Comparison of diversity estimates - species richness and evenness [Shannon-wiener (SW) diversity index]

**Fig. 2:**
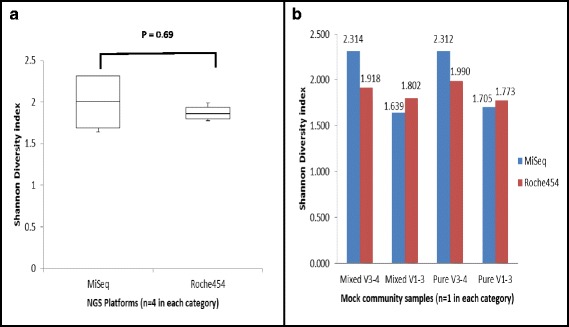
**a** Comparison of the alpha diversity estimates between NGS platforms for the mock bacterial communities. Box and whisker plot (median, interquartile range and min/maximum values) of the comparison of the Shannon Diversity index (y-axis) values obtained for the mock bacterial community at genus level classification for the two NGS platforms (x-axis) (*n =* 4) using Wilcoxon-rank sum test showed no significant difference between the two platforms (w = 10, *p =* 0.69). **b** Comparison of the alpha diversity estimates at genus level distribution for the primers and the NGS platforms for the mock bacterial community (MiSeq – blue column and Roche454 –red column –values indicated are Shannon diversity index in each category where *n =* 1). Mixed V3-4 and Mixed V1-3: Shannon diversity index for the two NGS platforms for the mock bacterial community with simulated human DNA contamination amplified using primer pair spanning V3-V4 and V1-V3 hypervariable regions of the 16S rRNA gene. Pure V3-4 and Pure V1-3: Shannon diversity index for the two NGS platforms for the mock bacterial community amplified using primer pair spanning V3-V4 and V1-V3 hypervariable regions of the 16S rRNA gene

The diversity at the phylum level was similar for both platforms. The mock bacterial community had an SW diversity index (*n =* 4 for each platform) of 1.85 and 2 for the Roche 454 Junior and the Illumina MiSeq platforms, respectively, when calculated based on the genus level distribution (Fig. 2a.). There was no significant difference observed between the two platforms (w = 10, *p =* 0.69) using the Wilcoxon-rank sum test, indicating that the MiSeq is comparable to the Roche 454 with respect to identifying the species richness and evenness in the bacterial mock community, at the genus level. The SW diversity index compared between platforms for the skin samples was also found to be similar with no significant difference observed (w = 14, *p =* 0.11). The primer pairs did, however, show within platform differences (Fig. 2b. SW index for the mock community samples on both platforms) for the Shannon diversity index values determined for the mock community samples (*n =* 1 for each category). The V3-4 primer pair captured more sequence diversity than the V1-3 primer pair on the MiSeq (w = 0, *p =* 0.03). The Roche 454 data indicated no such observed difference (w = 9, *p =* 0.89).

### Comparison of abundance of bacterial taxa between primer pairs and platforms

#### Data from bacterial mock community

At the phylum level, the mock bacterial communities showed similar ranking in abundance (Table 2) between the two platforms, with the three phyla in order of rank being *Firmicutes, Proteobacteria* and *Actinobacteria*. At this taxonomic level both the primer pairs performed similarly when assessed on the Roche 454 Junior platform, whereas the amplicons covering the V3-V4 hypervariable region on the MiSeq had closer concordance with the expected distribution of members of the mock community. Simulated contamination of the mock bacterial community with human DNA did not affect the taxonomic representation at any level on either platform.

**Table 2:** Abundance (%) of bacterial mock community at the level of phylum

Taxonomic level Phylum	Expected abundance (%)	MiSeq platform				Roche 454 GS Junior			
		V1-V3 primer pair		V3-V4 primer pair		V1-V3 primer pair		V3-V4 primer pair	
		MBx	MHx	MBx	MHx	MBx	MHx	MBx	MHx
Firmicutes	64	94.03	94.02	68.12	69.81	61.44	53.42	75.43	77.70
Proteobacteria	27	5.60	5.79	25.20	23.65	28.46	33.63	18.43	15.12
Actinobacteria	9	0.26	0.18	6.63	6.52	7.18	11.15	5.03	6.39

*MBx* In-house mock bacterial community with only bacterial DNA, *MHx* In-house mock bacterial community with human genomic DNA, *V1-V3 primer pair* primer pair targeting the V1-V3 hypervariable region of the 16S rRNA gene, *V3-V4 primer pair* primer pair targeting the V3-V4 hypervariable region of the 16S rRNA gene, *Roche 454 GS Junior platform* pyrosequencing next generation sequencing platform, *MiSeq platform* sequencing by synthesis next generation sequencing platform

**Fig. 3:**
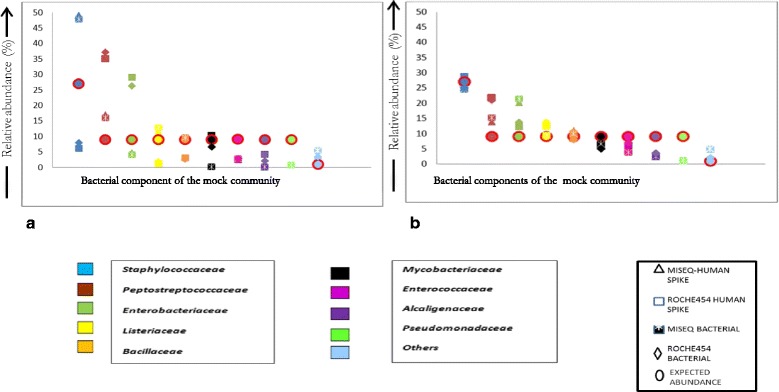
Pictorial representation of expected versus the actual relative abundance observed for the individual components of the mock bacterial community on the MiSeq and Roche454 GS Junior platform for primer pair targeting the (**a**) V1-V3 hypervariable region and (**b**) V3-V4 hypervariable region. Note: In the case of Pseudomonadaceae the MiSeq was able to identify the bacterial component at very low abundance <1.1% relative abundance for both primer pairs but this was not detected in the Roche454 data. (For the observed relative abundances see Additional file 1: Table S1)

Identification of bacterial taxa was most accurate at the family level for 9 of the 11 bacteria used in the mock community, on both NGS platforms (Fig. 3). No effect of the simulated human DNA contamination was observed, as replicates (with or without the simulated human DNA contamination) had very good concordance for the primer pairs when compared within each platform. When the primer pairs were compared between platforms, the V3-V4 primer pair performed better with values closer to the expected relative abundance of the mock community in comparison to that of the V1-V3 primer pair (Additional file 1: Table S1).

The V3-V4 primer pair demonstrated a maximum absolute difference of 7.96% and a minimum of 0.06% when compared between platforms. The V1-V3 primer pair however had a larger discrepancy with a maximum absolute difference of 41.7% and a minimum of 0.29% when comparing the relative abundances on the two platforms.

#### Skin swabs from healthy volunteers

Following quality control, data from three of the HV skin samples were compared between the two platforms for the primer pairs. Two of the skin samples were from the same individual for the V1-V3 and the V3-V4 hypervariable region. At the phylum level (Additional file 1: Table S2a) there was an identical distribution between the two platforms for the V3-V4 hypervariable region but less consistency for the V1-V3 hypervariable region.

At the genus level (Additional file 1: Table S2b), again the V3-V4 primer data showed better concordance between platforms than the V1-V3 primer pair.

### Comparison of beta diversity estimates of the mock bacterial community and the pilot data from skin swabs using procrustes PCoA plots

**Fig. 4:**
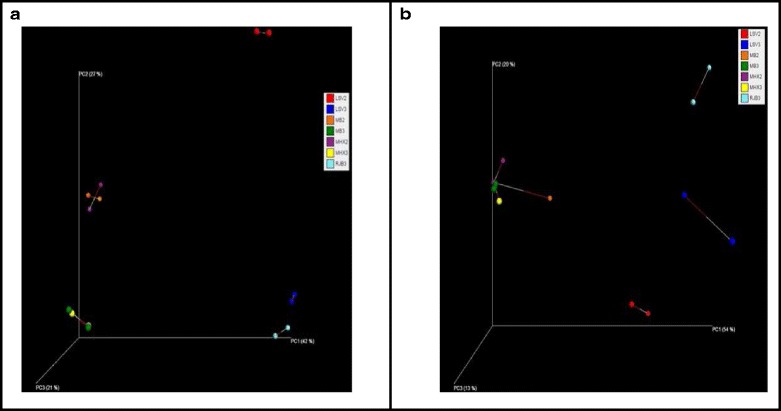
**a** Comparison of beta diversity results for the mock bacterial community and the healthy volunteer skin samples sequenced on both Roche 454 and Illumina MiSeq using Procrustes plot comparing the principal co-ordinates of Bray-Curtis distances. **b** Comparison of beta diversity results for the mock bacterial community and the healthy volunteer skin samples sequenced on both Roche 454 and Illumina MiSeq using Procrustes plot comparing the principal co-ordinates of Unweighted UniFrac distances. Lines connect paired sample sequences on the Roche454 (*white tip of line*) and Illumina Miseq (*red tip of line*)

The results of the Procrustes principle coordinates matrices are presented (Fig. 4a and Fig. 4b) with beta diversity estimates using the Unweighted Unifrac and the Bray Curtis distance matrices for visual representation of the diversity estimates. The mock bacterial communities with and without human DNA spiked into the samples and the three skin samples from healthy volunteers were available for analysis. The beta diversity for the mock bacterial community is identical for the V3-V4 primer pair when compared between the two platforms using the Unweighted UniFrac distance metric; however the V1-V3 primer pair shows more variability. By contrast, when using the Bray Curtis distance matrix which is based on abundance data for the beta diversity estimates, minimal variability is observed between platforms and between primer pairs. Similarly the beta diversity estimates for the human skin samples showed greater variability when evaluated using the Unweighted UniFrac for the two primer pairs on the two platforms when compared to the results from the Bray Curtis distance matrix.

## Discussion

In this paper we have described the development of a methodology for investigation of a very low biomass bacterial community using the MiSeq platform and compared data to that generated using the Roche 454 Junior system.

Comprehensive identification of any bacterial community is dependent on several steps including a robust bacterial DNA extraction method. A major concern with current protocols is their efficiency for extracting DNA from Gram positive bacteria, which have cell walls more resilient to lysis when compared to Gram negative bacteria [22, 23]. Given that Gram positive bacteria are commonly found on skin [24], namely *Staphylococcus,* a strength of the current study is that the bacterial DNA extraction method was first modified to ensure robust and reproducible capture of these species.

Previous studies [12, 13, 25] have compared the MiSeq and the 454 platforms but have used either mock bacterial communities [12, 13] or a single clinical sample [25]; by contrast, we have used both a mock community to show the accuracy of the V3-4 primer pair on the MiSeq platform when compared to the Roche454 and human samples to demonstrate that the primer pair will amplify sufficiently even in the low biomass environment of the skin.

The Microbiome Quality Control Project [26] recommends the use of a mock community containing taxa typical of site-specific microbial communities as a positive control in studies. Our work used the framework for human microbiome research [3] and included such a mock bacterial community to provide evidence for consistency and accuracy of the protocol. The mock community included a known skin commensal, Staphyloccocus species, which have been implicated in skin diseases such as atopic eczema where there is an increase in their abundance [27] and in psoriasis where reduced abundance has been reported [28]. In addition, the effect of simulated human DNA contamination was assessed. There have been studies on the effect of laboratory and reagent contamination in low biomass samples [10], on human-derived RNA-seq datasets [29] and bacterial contamination in human genome sequencing [30]. However, there is no information on the effect of human DNA contamination on the resulting output which is especially relevant in microbiome work on the skin as the bacterial biomass is low in comparison to the host genetic DNA that may contaminate the skin swab samples. Our work shows that the presence of human DNA does not affect the final output when compared to the results of the sequencing of the DNA from pure bacterial isolates increasing the confidence in the results obtained in human-derived samples. The data in the NIH Human Microbiome project [5] was derived from pyrosequencing using the Roche 454 FLX Titanium technology using amplicons generated from V1-V3 and V3-V5 hypervariable regions. The longer read lengths of 454 technologies cover a larger region of the 16S gene than the MiSeq and, therefore, enable easier taxonomic assignment. Shorter read lengths are sufficient to analyse microbial communities [11, 31], but limits the ability to maximize the capture of taxonomic diversity; therefore, we used the full capacity of the MiSeq with the 2x 250 bp paired end run (available at the time of the work) to achieve the optimal capture of data. Results demonstrated that the MiSeq performance was similar to the Roche 454 Junior platform in identifying community diversity for both the mock bacterial community and the skin samples at the phylum and the genus level.

Studies using the MiSeq platform have commonly used the V4 primer pair [11, 32] or the V3 primer pair [33], which limits the read length to ~250 bp. Our study demonstrates that the longer amplicons across the V3-V4 hypervariable region were identical for the abundance of the bacterial community identified and the beta diversity estimates when analysed on the MiSeq or Roche 454 Junior platforms. However, the MiSeq is more cost effective, as evidenced by the much higher coverage (>400x) obtained when compared to the Roche 454 Junior, using similar library preparation steps.

This work contributes to the evidence [12, 13, 34, 35] that the MiSeq is a suitable sequencing platform for profiling bacterial communities using analysis of 16S rRNA gene fragments. However, some limitations are apparent. We did not sequence negative controls which are considered as a measure of determining erroneous results [10] due to contamination as there was no detectable product in the samples. In our mock community we observed less than 5.5% in total abundance of other bacterial species and Rhizobium and Paracoccus, which have been reported as contaminating species in negative controls previously [10], showed abundances of less than 0.1%, lower than the traditional threshold (>1%) of abundance for inclusion in analysis. Another limitation of the current study reflects the rapidly evolving nature of microbiome research and the availability of advanced NGS with longer reads such as single molecule real time (SMRT) sequencing technologies which have not been explored or cross-validated. However, the results of this study show that the data generated by the MiSeq benchtop sequencer, which is now replacing the Roche454 platform in academic departments, is comparable. Therefore the data generated using the V3-V4 primer pair using the MiSeq can be compared with the existing data in the human microbiome literature.

Four of the eight skin samples showed high levels of chimera (~20%) on data analysis on the MiSeq platform and could not be confidently included in the comparative analysis between platforms. This is likely due to PCR artefacts introduced as a result of incomplete extension of the DNA template, or may be linked to the low starting biomass available, as well as the limitation of the sequence read length of the MiSeq. PCR based limitations are inherent in these analyses due to the need for amplification from low biomass samples and need to be taken into account when preparing libraries. Strategies to suppress the artificial chimera formation during PCR amplification have been reported [36–39] and could be used to minimize chimera generation during PCR. The loss of samples, though not anticipated at the outset of the work, has also been observed in a larger study investigating the skin microbiota in psoriasis [28]. Even in that study, the data from one of the primer pairs which targeted the V3-V5 hypervariable region could not be analysed as ~60% of the samples had to be removed due to amplification and sequencing depth quality issues even before the data analysis could be conducted. In the current work only one paired sample was lost due to sequencing depth issues and there were no losses due to PCR amplification. However, at the data analysis stage, four of the remaining skin swabs had high levels of chimeras detected and were therefore discarded from further analysis. This highlights the limitations that researchers should anticipate and try to mitigate against when studying low bacterial biomass samples. The mock community data could only be assigned to the family level in the taxonomic identification. This was considered to be the result of a combination of factors including the drop in quality of the MiSeq reads beyond 200 bps which resulted in a compromise in the quality of the 50 bp overlap, necessary for joining the paired end reads and the subsequent read/sequence assignment. It is expected that the upgraded version of the MiSeq chemistry, supporting the 300 bp paired end runs, will reduce the bias introduced due to inadequate read overlap at the sequencing stage and allow better taxonomic assignment. However, it was observed that in the HV skin samples, this was not a limiting factor as bacteria could be identified to the genus level despite using the version 2 chemistry on the MiSeq.

## Conclusions

Our study reports on the optimization of a protocol that can be used in investigating a low microbial biomass community, such as in skin samples. We have shown that the method is robust to spiking of human DNA into a low bacterial skin biomass mock community sample. Furthermore, we have demonstrated that the community diversity can be captured using the V3-V4 hypervariable region of the bacterial 16S rRNA gene for NGS and that the MiSeq is a suitable next generation sequencing platform, providing comparable data to the Roche 454 GS Junior, with similar capture of bacterial diversity, but with a much improved throughput and cost effectiveness. Therefore, our results provide evidence for validation of the MiSeq platform against the Roche454 using a known mock bacterial community containing typical skin bacteria and also evidence that the optimised method can be used in human clinical samples.

## Methods

### Aim, design and setting of study

The aim of the study was to optimise and validate the method for generation of the 16S rRNA library and compare the MiSeq against the 454 platform for the 16S rRNA data generated when using low bacterial biomass samples such as skin swabs to study the human bacterial microbiome.

### Subjects

Healthy volunteers (age range 30–50 years) were recruited to this study after obtaining informed verbal consent (MREC 99/8/84). They had no history of skin diseases or evidence of skin infections and had not used topical antibiotics, topical steroids or systemic antibiotics in the last 6 months. Healthy volunteers (*n =* 2: for initial validation of the MoBio Power Soil® DNA Isolation kit; *n =* 6: for optimization of PCR; *n =* 4: for initial ligation of adapters and index primers; *n =* 2: for resequencing of 16S rRNA amplicons on the MiSeq and the Roche 454 GS Junior platform) were identified and screened for any exclusion criteria (Additional file 1: Skin sampling) using a modification of the NIH HMP sampling protocol. The right and left antecubital fossae were sampled from each healthy volunteer labelled and processed separately.

### Sample collection

After obtaining consent, each individual was given written instructions about skin preparation prior to sampling and soap (without any anti-bacterial ingredient) was provided for hygiene needs for the two weeks before sampling. The sampling technique described in the NIH Microbiome manual of procedures [40] was used to collect the samples in an aseptic manner using Catch-All™ (Epicentre®) swabs pre-moistened with sample collection fluid (50mMol Tris with 1 mM EDTA and 0.5% Tween 20). To increase the yield of the skin bacterial flora the NIH protocol was modified to ensure that 4 cm^2^ area of skin at the antecubital fossa was swabbed. The swabs were inserted into, and transported in MoBio Power Bead® collection tubes and processed within 3 h of sampling. The work flow for the sample processing is illustrated in figure (Fig. 1).

### Method development

#### Selection of bacterial DNA extraction kit

The MoBio Power Soil® DNA Isolation kit was selected for use in this project as the kit is used as standard in the protocols followed by the NIH Human Microbiome Project [40].

#### Bacterial DNA extraction

Bacterial DNA extraction was carried out using the MoBio PowerSoil® DNA Isolation Kit protocol with the following modification. To increase the efficiency of the microbial cell lysis the duration of mechanical and chemical lysis was increased with introduction of a 15 min incubation period at 70 °C in the cell lysis process after the addition of MoBio buffer C1 solution. A final volume of 50 μl of elution buffer was used for recovery of bacterial DNA from columns following extraction.

The extracted DNA was quantified using the Qubit® dsDNA HS Assay fluorometric quantitation kit as per manufacturer’s instructions (Invitrogen, Life Sciences).

Eleven different bacterial isolates (from an in house collection) were inoculated onto standard growth media and incubated in an appropriate atmosphere at 37 °C for 18 h. DNA was extracted using the above method and was diluted with PCR grade water to the mean DNA concentration of the samples (~1 ng/μl). The mock community was prepared by combining equal volumes (10 μl) of the individual bacterial isolates. The concentration of the mock bacterial community was 1.42 ng/μl which was similar to the concentration of the DNA extracted from skin samples (1.2 ng/μl). This was done to ensure that the concentration of DNA template amplified in the downstream PCR reactions were uniform in all reactions.

### PCR and primers

#### Selection of primers

The consensus in the published literature favoured the inclusion of the V1-V4 hypervariable regions for bacterial identification as it captures the diversity of the bacterial microbiome when used on the 454 pyrosequencing platform. PCR primers were selected after optimisation of different PCR primer sets. Initial primer pairs (V1-V3: 63 F CAGGCCTAACACATGCAAGTC and 519R GTATTACCGCGGCAGCTGGCAC [18]; V3-V4 347 F GGAGGCAGCAGTRRGGAAT and 803R CTACCRGGGTATCTAATCC [19] and V4-V5: 517 F GCCAGCAGCCGCGGTAA and 926R CCGTCAATTYYTTTRAGTTT [19]) were selected based on the amplicon length (dictated by the capacity of the MiSeq 2x 250 paired end run to generate sequencing reads with a minimum overlap of 20 bp between the forward and the reverse read and for uniformity of product size ~450 bp) and product position (primers targeting the hypervariable regions across V1-V5 of the 16S rRNA gene that were used in the Human Microbiome Project [41] to generate data that could be compared against the Human Microbiome Database).

### Polymerase Chain Reaction for initial amplification of bacterial DNA

A 50 μl PCR reaction using NEBNext® High-Fidelity 2X PCR Master mix (New England Biolabs®) using the 5 ng-1 μg total DNA template reaction master mix protocol [25 μl of NEBNext® High-Fidelity 2X PCR Master Mix (New England Biolabs®), primer pair mix 20 μl (25 μM concentration) and 23 μl of extracted DNA] was assembled. The work was performed under a UV hood in DNA free cabinet and the PCR mix was immediately transferred to a thermocycler (M J Thermocycler) preheated to 98 °C. The PCR cycling conditions were 98 °C for 30 s, followed by 35 cycles of 98 °C for 10 s, annealing temperature of 60 °C for V1-V3 primer pair or 56 °C for V3-V4 primer pair for 30 s and 72 °C for 30 s, and 72 °C for 10 mins. These thermocycling conditions were based on the optimization work on the PCR.

### Bacterial DNA library preparation for MiSeq re-sequencing of 16S amplicons

The PCR products were purified using the Agencourt® AMPure® XP and quantified using the Qubit® Flurometric Quantitation (Life Sciences, Invitrogen) dsDNA HS Assay and found in the range of 1445 ng to 1925 ng of DNA products.

The NEBNext® Ultra® DNA Library Prep Kit for Illumina was used to prepare the DNA library as this kit has been optimised for low concentration (5 ng-1 μg) DNA input. This was ideal as the samples had total DNA input between 25-50 ng.

The resultant libraries were divided at this stage for the ligation of the specific adapters for the Illumina and the Roche 454 GS Junior platforms. The 454 pyrosequencing was carried out after cleanup following standard procedures at this step as the library was also barcoded here.

For the Illumina platform, the NEBNext® Ultra® DNA Library Prep Kit was used to barcode the adapter-ligated DNA as per protocol for 50 ng input DNA to enable multiplexing of the extracted bacterial DNA library. The 16S amplicon libraries were quantified using qPCR, normalized and pooled after determining the size of the amplicons using the Agilent Bioanalyser. 5 μl of the pooled 4nM DNA library was prepared for sequencing as per the MiSeq benchtop sequencer manufacturer’s protocol. As the DNA library was expected to have low diversity, the pooled samples were combined with denatured and diluted PhiX control (50% of the total volume). This resultant library was sequenced on the MiSeq Benchtop Sequencer to produce 2 x 250 paired end reads.

### Method validation

#### MoBio PowerSoil® DNA Isolation Kit Validation

Initial validation of the bacterial DNA extraction technique using the MoBio PowerSoil® DNA Isolation Kit was carried out using *Staphylococcus epidermidis* strain RP62A. This is a recalcitrant Gram positive bacterium, which is considered to be the most prevalent staphylococcal species in humans [42]. Two replicates of the extraction and blank extraction controls were simultaneously processed. A single band was identified in the extracted replicates on agarose gel electrophoresis. The DNA extraction protocol was modified during this process and this was validated by extracting bacterial DNA from eleven different strains of bacteria as described above.

### Quality control

#### To detect contamination

Simultaneously with each bacterial DNA extraction, a blank extraction kit control was conducted to ensure detection of any contaminants from the extraction kits. With each PCR reaction a negative control with no DNA template (volume substituted with PCR grade water) was also processed simultaneously for each primer pair to detect contamination from any source including PCR reagents. These control samples were processed through to the ligation of adapters and index primers. At each stage after amplification the products were quantified using Qubit® and visualized through agarose gel electrophoresis. No bands were visible on gel electrophoresis prior to the ligation of adapters and index primers and after the indexing of the library in the blank extraction kit control and negative (no DNA template) control for the PCR reactions.

#### To detect contamination from human DNA

During the optimization process to detect if contamination of the samples by host (human) DNA would produce erroneous results, varying proportions of extracted human DNA were added to the mixed positive controls and PCR amplification was undertaken using the selected primer sets. There was no amplification observed in the samples that were composed entirely of human DNA, demonstrating the specificity of the primers for bacterial targets.

#### To ensure validity of the kits and PCR reagents

A positive control (consisting of only one species of bacteria) was processed at every bacterial DNA extraction when working with skin samples, to ensure that the extraction kits and reagents were functional. In order to ensure that the protocol worked for every batch of the skin samples that were processed the positive control was also processed. At each stage after amplification the products were quantified using Qubit®, diluted to the same quantity as the samples and visualized using gel electrophoresis. Single bands were visualized at each gel electrophoresis that confirmed the presence of the appropriate PCR end product.

### Statistical data analysis

The microbial data was described using quantitative measures of community composition that take into account the presence and the abundance of a given taxa using the Shannon-Wiener alpha diversity index. To compare the alpha diversity estimates between the two NGS platforms and the primer pairs the non-parametric Wilcoxon rank sum test was used. For a visual comparison of the beta diversity estimates Procrustes analysis was carried out using the QIIME pipeline. The unweighted UniFrac and the Bray-curtis distance matrices were applied to the analysis to assess the beta diversity estimation on the two platforms for the qualitative (presence/absence of OTUs) and quantitative (abundance estimates) measures respectively when using the two different primer pairs on the two different sequencing platforms.

## Additional file

Additional file 1: Table S1. Comparison of the relative abundance (%) of bacterial mock community at the classification level of family. **Table S2**a. Comparison of the relative abundance (%) of bacterial taxa from healthy human skin samples at the phylum level (data available for two samples for the V3-V4 primer pair and one sample for V1-V3 primer pair). **Table S2**b. Comparison of the relative abundance (%) of bacterial taxa from healthy human skin samples at the genus level (data available for two samples for the V3-V4 primer pair and one sample for V1-V3 primer pair). **Skin sampling**: Requirements for skin sampling and skin preparation instructions provided to healthy volunteers. (DOCX 40 kb)

## Abbreviations

16S rRNA gene: 16S ribosomal ribonucleic acid – A component of the 30S small subunit of prokaryotic ribosomes
bp: Base pair
DNA: Deoxyribonucleic acid
Gb: Gigabyte
HV: Healthy volunteer
Mb: Megabyte
NCTC: National collection of type cultures
Q: Quality score eg: Q30 is Phred Score 30, 99.9% base call accuracy. I in 1000 probability of incorrect base call
V1-V3: Hypervariable region 1 through to 3 on the 16S rRNA gene
V3-V4: Hypervariable region 3 through to 4 on the 16S rRNA gene
V4-V5: Hypervariable region 4 through to 5 on the 16S rRNA gene
