# Supplementary material for: Optimisation of methods for bacterial skin microbiome investigation: primer selection and comparison of the 454 versus MiSeq platform
Source: BMC Microbiol. 2017 Jan 21;17:23. doi: 10.1186/s12866-017-0927-4 (PMC5251215; doi:10.1186/s12866-017-0927-4)
Supplement: Additional file 1: Table S1. — Comparison of the relative abundance (%) of bacterial mock community at the classification level of family. Table S2a. Comparison of the relative abundance (%) of bacterial taxa from healthy human skin samples at the phylum level (data available for two samples for the V3-V4 primer pair and one sample for V1-V3 primer pair). Table S2b. Comparison of the relative abundance (%) of bacterial taxa from healthy human skin samples at the genus level (data available for two samples for the V3-V4 primer pair and one sample for V1-V3 primer pair). Skin sampling: Requirements for skin sampling and skin preparation instructions provided to healthy volunteers. (DOCX 40 kb) [file 12866_2017_927_MOESM1_ESM.docx]

Supplementary Information

Supplementary Table S1: Comparison of the relative abundance (%) of bacterial mock community at the classification level of family

| **Taxonomic level - Family** | **Expected abundance**  **(%)** | **MiSeq platform**  **Relative bacterial abundance identified (%)** | | | | **Roche 454 GS Junior**  **Relative bacterial abundance identified (%)** | | | |
| --- | --- | --- | --- | --- | --- | --- | --- | --- | --- |
|  |  | V1-V3 primer pair | | V3-V4 primer pair | | V1-V3 primer pair | | V3-V4 primer pair | |
| **Mock community constituents** | Expected | MBx | MHx | MBx | MHx | MBx | MHx | MBx | MHx |
| Staphylococcaceae | 27 | 47.82 | 49.00 | 24.66 | 26.42 | 7.98 | 6.12 | 25.09 | 28.78 |
| Peptostreptococcaceae | 9 | 16.06 | 17.11 | 15.23 | 13.87 | 37.23 | 35.07 | 21.00 | 21.67 |
| Enterobacteriaceae | 9 | 4.18 | 4.86 | 21.46 | 20.19 | 26.33 | 29.14 | 13.74 | 12.23 |
| Listeriaceae | 9 | 12.84 | 11.65 | 9.51 | 9.74 | 1.86 | 1.26 | 13.23 | 12.41 |
| Bacillaceae | 9 | 9.71 | 9.67 | 10.06 | 11.18 | 2.93 | 3.06 | 8.02 | 8.36 |
| Mycobacteriaceae | 9 | 0.13 | 0.12 | 6.53 | 6.41 | 6.65 | 10.43 | 5.03 | 6.39 |
| Enterococcaceae | 9 | 2.81 | 2.51 | 3.90 | 3.95 | 2.93 | 2.52 | 7.00 | 6.03 |
| Alcaligenaceae | 9 | 0.13 | 0.11 | 2.57 | 2.37 | 2.13 | 4.32 | 3.67 | 2.43 |
| Pseudomonadaceae | 9 | 0.83 | 0.78 | 1.13 | 1.05 |  |  |  |  |
| Others | 0 | 5.49 | 4.20 | 4.97 | 4.85 | 3.46 | 1.98 | 2.13 | 0.90 |

MBx: In-house mock bacterial community with only bacterial DNA; MHx: In-house mock bacterial community with human genomic DNA simulating human DNA contamination; V1-V3 primer pair: primer pair targeting the V1-V3 hypervariable region of the 16S rRNA gene; V3-V4 primer pair: primer pair targeting the V3-V4 hypervariable region of the 16S rRNA gene; Roche 454 GS Junior platform – pyrosequencing next generation sequencing platform; MiSeq platform – sequencing by synthesis next generation sequencing platform

| **Taxonomic level** | **MiSeq platform** | | | **Taxonomic level** | **Roche 454 GS Junior** | | |
| --- | --- | --- | --- | --- | --- | --- | --- |
|  | **Data for**  **V3-V4**  **primer pair** | | **Data for V1-V3 primer pair** |  | **Data for**  **V3-V4**  **primer pair** | | **Data for V1-V3 primer pair** |
| **Phylum** | **HC1: Right ACF** | **HC2: Left ACF** | **HC2: Left ACF** | **Phylum** | **HC1: Right ACF** | **HC2: Left ACF** | **HC2: Left ACF** |
| Actinobacteria | 32.85 | 42.8 | 45.14 | Actinobacteria | 27.50 | 40.22 | 51.6 |
| Proteobacteria | 23.45 | 29.79 | 29.56 | Proteobacteria | 26.44 | 41.80 | 41.05 |
| Firmicutes | 19.40 | 23.88 | 24.00 | Firmicutes | 19.29 | 14.64 | 2.64 |
| Bacteroidetes | 9.87 | 0.74 | 0.44 | Bacteroidetes | 9.99 | 0.45 | 0 |
| Thermi | 6.91 | 0.12 | 0.04 | Thermi | 5.97 | 0 | 0 |
| Cyanobacteria | 4.65 | 1.82 | 0.46 | Cyanobacteria | 5.04 | 1.34 | 0.57 |
| TM7 | 1.38 | 0.23 | 0.12 | TM7 | 2.40 | 0 | 0 |
| Other | 1.49 | 0.62 | 0.24 | Other | 2.64 | 1.56 | 4.14 |

Supplementary Table S2a: Comparison of the relative abundance (%) of bacterial taxa from healthy human skin samples at the phylum level (data available for two samples for the V3-V4 primer pair and one sample for V1-V3 primer pair)

HC1: Healthy control 1; HC2: Healthy control 2; ACF: Antecubital fossa; V1-V3 primer pair: primer pair targeting the V1-V3 hypervariable region of the 16S rRNA gene; V3-V4 primer pair: primer pair targeting the V3-V4 hypervariable region of the 16S rRNA gene, Roche 454 GS Junior platform – pyrosequencing next generation sequencing platform, MiSeq platform – sequencing by synthesis next generation sequencing platform

Supplementary Table S2b: Comparison of the relative abundance (%) of bacterial taxa from healthy human skin samples at the genus level (data available for two samples for the V3-V4 primer pair and one sample for V1-V3 primer pair)

| **Taxonomic level** | **MiSeq platform** | | | **Taxonomic level** | **Roche454 GS Junior platform** | | |
| --- | --- | --- | --- | --- | --- | --- | --- |
|  | **V3-V4 primer pair** | | **V1-V3 primer pair** |  | **V3-V4 primer pair** | | **V1-V3 primer pair** |
| **Genus** | **HC1: Right ACF** | **HC2: Left ACF** | **HC2: Left ACF** | **Genus** | **HC1: Right ACF** | **HC2: Left ACF** | **HC2: Left ACF** |
| *Staphylococcus* | 11.62 | 12.47 | 20.49 | *Staphylococcus* | 15.48 | 8.27 | 1.32 |
| *Propionibacterium* | 8.05 | 20.29 | 20.74 | *Propionibacterium* | 7.64 | 21.34 | 32.39 |
| *Corynebacterium* | 8.87 | 12.77 | 18.88 | *Corynebacterium* | 6.70 | 10.61 | 9.79 |
| *Paracoccus* | 1.94 | 6.97 | 7.75 | *Paracoccus* | 3.17 | 11.06 | 13.18 |
| *Micrococcus* | 2.76 | 4.53 | 2.62 | *Micrococcus* | 1.99 | 4.69 | 6.21 |
| *Enhydrobacter* | 1.70 | 6.51 | 0.42 | *Enhydrobacter* | 2.80 | 9.94 | 3.58 |
| *Deinococcus* | 6.91 | 0.02 | 0 | *Deinococcus* | 5.97 | 0 | 0 |
| *Rosemonas* | 0.20 | 5.16 | 10.17 |  |  | . |  |
| *Other* | 49.39 | 22.32 | 13.86 | *Other* | 50.24 | 20.11 | 16.38 |

HC1: Healthy control 1; HC2: Healthy control 2; ACF: Antecubital fossa; V1-V3 primer pair: primer pair targeting the V1-V3 hypervariable region of the 16S rRNA gene; V3-V4 primer pair: primer pair targeting the V3-V4 hypervariable region of the 16S rRNA gene, Roche 454 GS Junior platform – pyrosequencing next generation sequencing platform, MiSeq platform – sequencing by synthesis next generation sequencing platform

**Supplementary information Skin sampling**

Healthy volunteers recruited to the initial phase of the pilot work did not have any evidence of arthritis or skin disease. The volunteers were recruited opportunistically from the University of Manchester who did not have the following exclusion criteria.

- Use of antibiotics or any active medications within the preceding 6 months
- Local skin lesions, previous history of skin disorders, clinical evidence of any skin lesion
- Use of topical treatment within the last 3 months

The skin sampling technique was based on the NIH HMP manual of procedures (NIH HMP Manual of Procedures Protocol A HMP Protocol #07-001 v12). Each subject was given written instructions about skin preparation prior to sampling. Soap and shampoo were provided for hygiene needs for a period of two weeks prior to the sampling.

The identified individuals were clinically examined for any current active skin lesions. To reduce variation due to use of hygiene products, volunteers were provided with soap and instructed to use it for 2 weeks prior to sampling. They were provided with a leaftlet (shown below) with instructions on the activities they needed to avoid for successful sampling .

**SKIN PREPARATION INSTRUCTIONS**

Date of scheduled sampling visit: ______________________.

**Three months before sample collection visit**

- No antibiotic, antifungal, antiviral or antiparasitic drugs; by mouth, by injection or intravenously.
- No commercial probiotics in doses greater than or equal to 10^8^ (cfu) or organisms per day, including tablets, capsules, lozenges, chewing gum or powders in which probiotic is a primary component. Note that it is acceptable to consume foods such as yogurt and fermented beverages/milks.

**Seven (7) days before sample collection visit:**

Date to stop using products: ____________________

- No antibiotics or steroids applied as creams or ointments on the skin of the face, scalp, neck, arms, forearms or hands.

**48 hours before sample collection visit:**

Date and time to stop activities and use of products: __________________

- No antimicrobial products including liquid hand soap, bar soap, face washes and hand or mouth washes.
- No antiseptic products such as hand or mouth washes, perfumes and sanitizers (such as Listerine mouth wash and Purell hand wash)
- No hair dyes of any kind
- No use of a chlorinated pool or hot tub

Date and time to start using personal care products provided: ________________

**12 hours before scheduled sample collection visit:**

Date and time to stop activity: _____________

- No showering and bathing. Note that hand washing with soap provided in the personal care kit is allowable.

**You will be provided with a kit that contains personal care products that are acceptable for use during the 48 hours prior to sampling. If you wish to use alternative products, please consult the following list of basic personal care products that do not contain antibacterial agents.**

| **Product** | **Please use the following:** |
| --- | --- |
| **Soap** | Camay Bar Soap (Procter & Gamble [P&G])  Ivory Classic Bar Soap (P&G)  Olay Bar Soap (P&G)  Simple Soap |
| **Skin moisturizer** | Aquaphor Healing Ointment (Eucerin)  Johnson’s Baby Oil (Johnson & Johnson [J&J])  White Petrolatum (Vaseline) |
| **Shampoo** | Pantene Clarifying Shampoo (P&G)  Pert Plus Deep Moisturizing Shampoo Plus Conditioner for Normal Hair (P&G) |
| **Conditioner** | Pantene Pro-V Sheer Volume conditioner or Pantene Pro-V Daily Moisture Renewal conditioner (P&G) |
| **Mouth wash** | Homemade rinse of ½ tsp salt or ½ tsp baking soda in 8 ounces of water. |

**Please avoid any products with the following ingredients in:**

| **Ingredient Group** | **Ingredient** |
| --- | --- |
| Anilides | triclocarban |
| Biguanides | Chlorhexidine  Alexidine  Polymeric biguanides |
| Bisphenols | Triclosan |
| Halophenols | PCMX (p-chloro-m-xylenol) |
| Phenols and cresols | Phenol  Cresol  Thymol |
| Quaternary ammonium compounds | Cetrimide  Benzalkonium chloride  Cetylpyridinium chloride |
